# When the MOUSE leaves the house

**DOI:** 10.5194/mr-2-149-2021

**Published:** 2021-04-16

**Authors:** Bernhard Blümich, Jens Anders

**Affiliations:** 1 Institut für Technische und Makromolekulare Chemie, RWTH Aachen University, 52159 Roetgen, Germany; 2 Institute of Smart Sensors, University of Stuttgart, 70569 Stuttgart, Germany

## Abstract

Change is inherent to time being transient. With the NMR-MOUSE (MObile Universal Surface Explorer) having matured into an established NMR tool for nondestructive testing of materials, this forward-looking retrospective assesses the challenges the NMR-MOUSE faced when deployed outside a protected laboratory and how its performance quality can be maintained and improved when operated under adverse conditions in foreign environments. This work is dedicated to my dear colleague and friend Geoffrey Bodenhausen on the occasion of his crossing an honorable timeline in appreciation of his ever-continuing success of fueling the dynamics of magnetic resonance.

## 1 Introduction

The MOUSE (MObile Universal Surface Explorer) (Eidmann et al., 1996) is a portable stray-field NMR sensor suited for nondestructive testing of materials (Blümich et al., 2008; Casanova et al., 2011). With it, the relaxation of nuclear spins towards equilibrium is measured following perturbation by radio-frequency (rf) pulses. The sensor is a small and compact NMR relaxometer that investigates an object from one side and can be carried along to the site of interest. As such the NMR-MOUSE and other stray-field relaxometers are one modality of compact NMR. Other modalities are tabletop relaxometers, tabletop imagers, and tabletop spectrometers (Blümich et al., 2014; Blümich, 2016; Blümich and Singh, 2018).

While the size of most NMR instruments today is dominated by a large superconducting magnet, compact NMR relaxometers have small permanent magnets. They were commercially introduced in the early 1970s to assist the food industry in characterizing emulsions (van Putte and van den Enden, 1974; Blümich, 2019a). Today tabletop relaxometers are employed to study a wide range of materials, in particular foodstuffs, polymers, and porous media (Blümich et al., 2014; Blümich, 2016; Blümich and Singh, 2018; Saalwächter, 2012). A key feature of these early tabletop instruments is that samples need to be drawn and inserted into a hole in the magnet for analysis. In this respect, the measurement is destructive. This equally applies to modern tabletop NMR spectrometers for chemical analysis unless they are operated in flow-through mode, like in a process control environment (Kern et al., 2019). While NMR spectrometers with permanent magnets were built already in the early 1950s (Gutowsky et al., 1953; Blümich, 2019a), their magnets were large and could produce only a small field region sufficiently homogeneous to resolve the proton chemical shift. Small permanent magnets with homogeneous fields are challenging to build due to the variations in dimensions, polarization magnitude, and direction of the magnet elements. Therefore, the routine use of compact NMR instruments remained limited for a long time to relaxation and diffusion measurements until the technology of compact high-resolution magnets had been sufficiently advanced about 10 years ago (Danieli et al., 2010; Blümich, 2016; Blümich and Singh, 2018). Before that, chemical analysis with tabletop instruments was explored primarily by a few dedicated research groups (Nordon et al., 2001; Dalitz et al., 2012).

Mobile NMR instruments need to be both compact and robust to deploy them at different sites and in different environments. The era of mobile NMR began with well-logging instruments shortly after the first NMR experiments in condensed matter in December 1945. Already in 1952, Russell Varian patented a subsurface well-logging method and apparatus (Varian, 2021; Woessner, 2001). The sensor to be inserted into the borehole of an oil well and operating in the earth's magnetic field eventually evolved into tube-shaped instruments housing permanent magnets as well as transmit and receive electronics to analyze the relaxation of ${}^{1}$ H NMR signals from particular regions localized in the borehole wall (Jackson et al., 1980; Kleinberg and Jackson, 2001). In his introduction to the 2016 book *Mobile NMR and MRI* (Johns et al., 2016), Eiichi Fukushima reviews the evolution of earth-field and mobile NMR with particular attention to these early developments (Fukushima, 2016).

Well-logging NMR is also known as inside-out NMR, because the instrument is inserted into the object and not the object into the magnet. Inside-out NMR is mobile but also destructive, as a hole needs to be drilled into the object (Jackson et al., 1980; Coates et al., 1999). The underlying concept of mobile stray-field relaxometry was extended at the Southwest Research Institute, San Antonio, Texas, to nondestructive materials testing with NMR relaxometers accessing the object from one side. These instruments were already transportable, whereby some of them used bulky and massive electromagnets and others used more compact permanent magnets (Fukushima, 2016). The magnets were laid out to maximize the field volume containing the spins which can be excited selectively from within the bulk with rf pulses in an effort to maximize the hydrogen signal from the object of interest next to the sensor deriving from moisture in soil, bridge decks, building structures, and food products (Fukushima, 2016; Blümich et al., 2008; Blümich, 2016, 2019b). Within this volume, the field gradient must be small enough so that the resonance frequencies of the spins inside are within the bandwidth of the rf excitation pulses. As a consequence, the field strength was low.

One may argue that the era of mobile NMR with compact sensors essentially started with the appearance of the NMR-MOUSE, a stray-field relaxometer that in its design disregarded the quest for a large sensitive volume by fortuitous ignorance (Eidmann et al., 1996). The small sensor exhibits a large field gradient and consequently a small sensitive volume yet a strong stray field. Compared to larger sensors, the opposing impacts on the sensitivity of a smaller signal-bearing volume and higher field strength turned out to largely balance each other so that at comparable sensitivity the more compact sensors (Blümich et al., 2008) were easier to carry along and be moved from one place to another than other stray-field sensors.

## 2 The NMR-MOUSE in the house

While brainstorming the simplest realization of NMR in 1993 at the Max Planck Institute for Polymer Research in Mainz, Peter Blümler asked the following question: “Would it not be nice to have an NMR scanner that one moves across the surface of an object to look inside just like an ultrasound scanner?” (Armstrong-Smith, 2015). The next day he came with a drawing of how such a device could look like, and we dubbed it NMR-MOUSE for “MObile Universal Surface Explorer”. Having taken up the position of Chair of Macromolecular Chemistry at RWTH Aachen University the same year, the realization of the NMR-MOUSE was the project of Blümich's first PhD student Gunnar Eidmann at RWTH Aachen University, who succeeded to get the first signal in 1995 (Eidmann et al., 1996). Hardware improvements, measurement methodology, and applications of the NMR-MOUSE were systematically explored over the years in particular by Peter Blümler, Gisela Guthausen, Sophia Anferova, Valdimir Anferov, Federico Casanova, and Juan Perlo. The NMR-MOUSE has found numerous applications for nondestructive materials characterization by relaxation and diffusion measurements (Blümich et al., 2008; Casanova et al., 2011). The design of many early stray-field relaxometers and of the original NMR-MOUSE was that of a simple U-shaped magnet. It is marketed by Bruker under the name “minispec ProFiler”. This sensor has a roughly cup-shaped sensitive volume, the position and shape of which are defined by the profiles of the stray fields produced by the permanent magnet and the radio-frequency coil (Eidmann et al., 1996; Hürlimann and Griffin, 2000; Balibanu et al., 2000).

**Figure 1:**
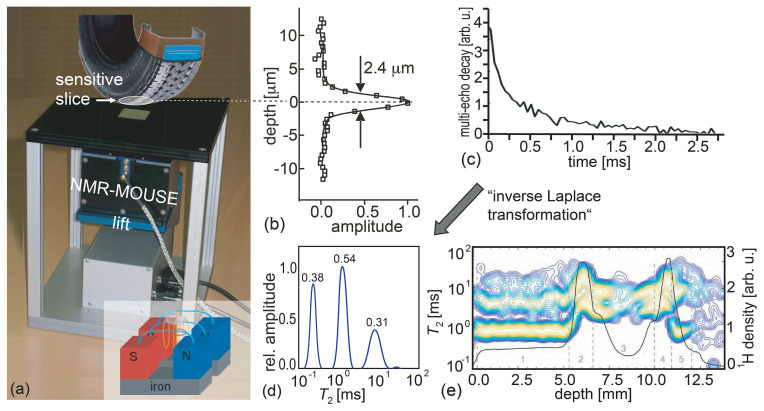
The principle of measuring depth profiles with the Profile NMR-MOUSE. **(a)** Conceptual picture of the Profile NMR-MOUSE on a lift with its sensitive slice inside a rubber tire. **(b)** Point-spread function of the record depth resolution. **(c)** Experimental signal decay measured with a multi-echo train. **(d)** Distribution of relaxation times from tire-tread rubber derived by inversion of a signal decay with an algorithm, referred to as “inverse Laplace transformation”. **(e)** Collection of distributions of relaxation times and signal amplitudes reporting nominal spin density measured across a range of 14 mm into a tire tread.

A major improvement of the original sensor was to shim the sensitive volume from a bowl shape to a flat slice with a diameter of about 10 mm and, depending on the measurement scheme, a slice width of less than 3 µm (Perlo et al., 2005a), enabling the acquisition of high-resolution depth profiles by translating the sensor in-between measurements with high precision. To this end, two U-shaped or horseshoe magnets are placed side by side with a small gap (Fig. 1a, bottom). The measurement principle followed to acquire depth profiles is the same as that employed for logging oil wells except that the NMR-MOUSE sensor is horizontally moved between consecutive measurements in steps on the order of 0.1 mm instead of acquiring NMR signal while the well-logging tool is moving laterally with respect to the magnet surface for distances on the order of meters (Coates et al., 1999; Hürlimann and Heaton, 2016). Today, the NMR-MOUSE for high-resolution depth profiling is a heritage product of Magritek GmbH with its production site in Aachen, which is managed by the two NMR-MOUSE pioneers Federico Casanova and Juan Perlo. In fact, Magritek today is the result of a 2012 merger of Magritek Ltd. from New Zealand, which, among others, developed the Kea spectrometer motivated by Paul Callaghan's Antarctic expeditions (Callaghan et al., 1998), and ACT GmbH, a company spun off from RWTH Aachen University, which produced the “Profile NMR-MOUSE”.

To measure depth profiles, the sensor is mounted on a precision displacement stage with which the sensitive slice at a fixed distance from the magnet surface can be moved through the object step by step between acquisitions of multi-echo trains and more advanced two-dimensional Laplace methods (Song et al., 2002; Callaghan, 2011; Blümich et al., 2014). The envelope of a multi-echo train provides a stroboscopically sampled transverse relaxation decay (Fig. 1c) from which a depth-profile amplitude can be derived in different ways to provide NMR parameter contrast based on the relaxation-time distribution (Fig. 1d), the hydrogen density corresponding to the signal amplitude from the spins in the sensitive slice (Fig. 1e), relaxation times, and molecular self-diffusion (Blümich et al., 2008; Casanova et al., 2011; Blümich et al., 2014). The signal amplitude is the full integral of the relaxation-time distribution. Partial integrals of individual peaks provide component concentrations. Assigning physical meaning to individual peaks is not as straightforward as interpreting the chemical shift of resonance lines in a high-resolution NMR spectrum. Yet the peak amplitudes and positions vary with material properties (Fig. 1e), and it often takes the richness of experience or a reference database to interpret distributions of relaxation times for practical applications.

**Figure 2:**
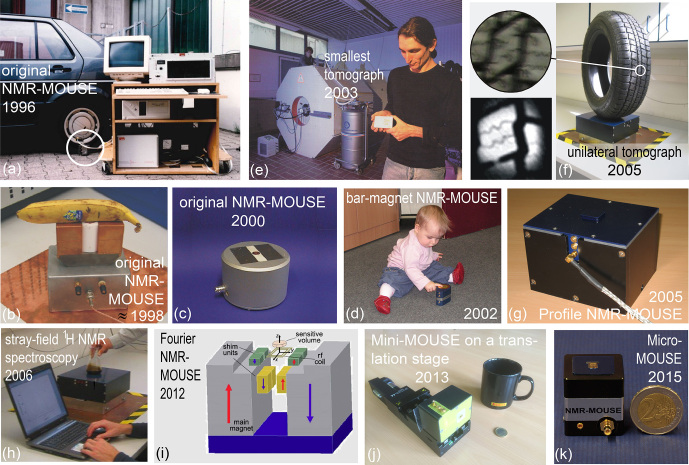
The evolution of stray-field NMR at RWTH Aachen University. **(a)** The original NMR-MOUSE measuring a car tire. **(b)** An early version of the NMR-MOUSE with copper-shielded magnets for dead-time reduction. **(c)** The original NMR-MOUSE in 2000. **(d)** The bar-magnet NMR-MOUSE is the simplest construction of a stray-field NMR sensor. **(e)** In 2003 Juan Perlo developed the smallest tomograph in stray-field technology (photo: Peter Winandy). **(f)** A single-sided tomograph with a flat imaging plane. Right: setup for mapping a tire tread. Left: a photo (top) in comparison with an MR image (bottom). **(g)** The Profile NMR-MOUSE with a flat sensitive slice developed in 2005. **(h)** Stray-field NMR magnet capable of measuring chemical-shift-resolved ${}^{1}$ H NMR spectra from a fluid in a beaker placed on top of the magnet. **(i)** Fourier NMR-MOUSE with shim magnets producing a 2 mm thick sensitive slice for frequency encoding of depth. **(j)** Mini-MOUSE with a multilayered micro-coil having a dead time of 10 µs . **(k)** Micro-MOUSE constructed from four 1 cm ${}^{3}$ permanent magnet cubes.

The hardware, use, and measurement methodology of the NMR-MOUSE has been studied for more than 2 decades in various research projects at RWTH Aachen University and other places. Its use for testing different materials such as rubber, polymers, building materials, food, and objects of cultural heritage is reported in books and reviews (Blümich, 2000, 2008; Blümich et al., 2008, 2010; Casanova et al., 2011; Capitani et al., 2012; Blümich et al., 2014; Baias and Blümich, 2017; Capitani et al., 2017; Blümich, 2019c; Rehorn and Blümich, 2018). Several modifications of the original NMR-MOUSE in addition to the forerunner of the current Magritek Profile NMR-MOUSE (Fig. 1a) have been investigated at RWTH Aachen University. Recognizing that the information content extractable from the signal of the sensitive region in stray-field NMR corresponds to that accessible in a voxel of a magnetic resonance image; first applications of the NMR-MOUSE were explored with car tires representing soft synthetic matter (Fig. 2a) in line with the main use of MRI in imaging soft biological matter. The horseshoe setup (Fig. 2b) was subsequently made smaller and packed into a more attractive shell (Fig. 2c). Realizing that the horseshoe magnet, having the $B_{0}$ stray field essentially parallel to its active surface, could be further simplified, the bar-magnet NMR-MOUSE was built from a magnet block first cuboid shaped (Blümich et al., 2002a) and later cylinder shaped with $B_{0}$ perpendicular to the active end face and a figure-eight rf coil with $B_{1}$ parallel to it (Fig. 2d). The maximum depth of access is lower than for the horseshoe sensor, but the dead time is shorter due to the gradiometer property of the rf coil (Anferova et al., 2002). Note that contrary to the $B_{0}$ orientation perpendicular to the active surface, $B_{0}$ parallel to the active surface enables studying macroscopic molecular order in anisotropic materials such as tendon and strained rubber (Haken and Blümich, 2000; Hailu et al., 2002).

While improving the original NMR-MOUSE, a single-sided tomograph was also developed and tested in the Deutsche Forschungsgemeinschaft (DFG)-funded Collaborative Research Center on “Surface NMR of Elastomers and Biological Tissue” FOR333. One result from this project was the smallest MRI instrument at the time, which was obtained by fitting a bar-magnet NMR-MOUSE with coils for pulsed gradient fields (Fig. 2e) (Casanova and Blümich, 2003). Another result was an improved U-shaped magnet with thicker ends at each side of the poles so that the magnet assembly produced a flat imaging plane (Fig. 2f) (Casanova and Blümich, 2003; Blümich et al., 2005). Images from a plane parallel to the sensor surface could be measured with pure phase-encoding schemes, but the sensitivity was low due to the thin slice resulting from a strong stray-field gradient. Maintaining the flat sensitive region of the imaging plane, this complex magnet geometry was subsequently simplified to two U-shaped magnets placed at a specific distance next to each other, resulting in the Profile NMR-MOUSE (Figs. 1a, 2g), which proved to be a robust stray-field NMR sensor constructed from a minimum number of parts (Perlo et al., 2005a).

Flattening the sensitive region of a stray-field magnet to a plane was a milestone in understanding how to shim the stray field. Eventually, the sensitive region in the stray field could be homogenized locally with the help of additional shim magnets to a degree sufficient to resolve the ${}^{1}$ H chemical shift from a limited volume of fluid inside a beaker on top of the magnet (Fig. 2h) (Perlo et al., 2005b, 2006; Zalesskiy et al., 2014). Another advance was the construction of a stray-field sensor with a sensitive slice having a homogeneous gradient field in the sensitive slice across an extended depth range of 2 mm for single-shot depth profiling by frequency encoding of position (Fig. 2i) (Van Landeghem et al., 2012). With this sensor the time to acquire a depth profile into soft matter was reduced considerably, and it proved useful for in vivo applications like mapping human skin and monitoring perfusion states of the small intestine by diffusion maps (Keschenau et al., 2018).

**Figure 3:**
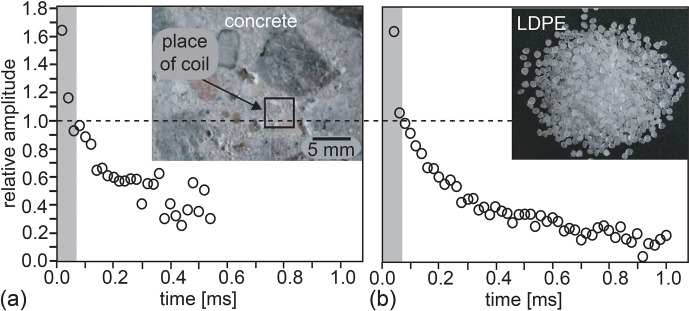
Transverse magnetization decays acquired with the Mini-MOUSE (Fig. 2j) at short echo time $t_{E}$ . The shaded area marks signal lost at a dead time of 70 µs . **(a)** The signal from bound water in dry, gray cement. **(b)** The crystalline protons in low-density polyethylene with $T_{2}=12$ µs can be detected at $t_{E}=20$ µs .

The NMR-MOUSE has also been miniaturized and fitted with multilayered micro-coils (Fig. 2j, k) (Watzlaw et al., 2013; Oligschläger et al., 2014, 2015a), by which, on the expense of 7.6-fold lower sensitivity compared to the PM5 NMR-MOUSE at 1 mm access depth, the dead time of the measurement could be reduced to a record 10 µs echo time. With its small coil, the signal from the cement regions between the stone aggregate in cuts of concrete could be focused on, and with an echo time ≤20 µs , the hitherto hidden signals from bound water in the dry gray cement could be measured (Fig. 3a) (Oligschläger et al., 2015a). At the same echo time, even the rapidly relaxing transverse magnetization from the crystalline domains of polyethylene was detectable (Fig. 3b). The presence of the rapidly decaying signal components at $t_{E}=20$ µs nearly doubles the amplitude of the recorded magnetization decays compared to the amplitudes recorded with the minimum echo time of 70 µs of the reference laboratory NMR-MOUSE with 10 mm depth of access. As the short dead time of the mini-MOUSE had been achieved at the cost of a small sensitive volume and a low depth of access due to the small diameter of the coil, it is the ambition of current sensor improvement to reduce the dead time at coil diameters 10 mm and more. Currently, for example, the minimum echo time of the Magritek PM25 NMR-MOUSE is better than 50 µs when fitted with spacers and a 15 mm diameter coil to limit the depth of access to 10 mm. For a new PM2 NMR-MOUSE with 2 mm depth of access, the minimum echo time is just 15 µs . Even shorter echo times may eventually be realized with novel transceiver circuits that promise the detection of the spin response during the rf pulse (Anders and Lips, 2021). Moreover, to shorten the acquisition time from hours to minutes for field applications like investigations of glass-fiber- or carbon-fiber-reinforced polymer materials employed in windmill wings and airplane rudders, the detection of the bitumen component in asphalt (Blümich et al., 2019), and the bound water in cement, a large sensitive volume is needed. This can be achieved, for example, with a coil array (Oligschläger et al., 2015b) placed on a suitably tailored magnet surface (Blümich et al., 2002b).

## 3 The NMR-MOUSE outside the house

The NMR-MOUSE was introduced to the cultural heritage community through the effort of Annalaura Segre at the turn of the millennium (Segre and Blümich, 2002), and from 1999 to 2019 its further refinement has benefitted greatly from the cultural heritage projects EUREKA-Eurocare Σ !2212-MOUSE, EU-ARTECH, CHARISMA, and IPERION-CH. With the exception of well-logging relaxometry (Coates et al., 1999), it is common practice to conduct NMR measurements in a laboratory. But objects of cultural heritage often cannot leave the museum or are immobilized, e.g., at excavation sites, so the NMR-MOUSE has to be moved to the site and operated under the prevailing environmental and climatic conditions. These aspects can be rather challenging at times for the operators as well as for the equipment, which has been designed primarily for indoor use.

**Figure 4:**
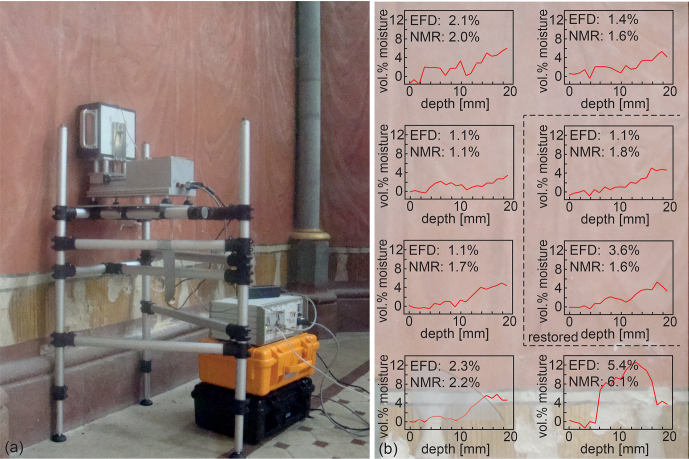
Moisture measurements at the painted wall left of the altar inside the Chapel of St. Mary in Chaalis. **(a)** Setup for NMR depth profiling. **(b)** High-resolution depth profiles from eight positions. The moisture-content values from evanescent field dielectrometry (EFD) are compared to NMR values derived from integration of quantitative moisture depth profiles weighted with the heuristic depth attenuation function, which is indicated by the shaded region in the bottom left profile. The background shows the approximate wall positions of the measurements.

For some outdoor applications like determining the crumb-rubber content in asphalt pavements (Blümich et al., 2019), depth profiling is not essential. But for others it is crucial. This includes the analysis of easel paintings (Presciutti et al., 2008; Fife et al., 2015; Angelova et al., 2016; Prati et al., 2019; Busse et al., 2020), frescoes (Rehorn et al., 2018), and mummies (Rühli et al., 2007; Blümich et al., 2014). A less obvious application is the analysis of moisture distributions, for example, in walls. Moisture maps with crude lateral resolution and high depth resolution can assist in locating a moisture leak (Proietti et al., 2007; Rehorn and Blümich, 2018; Blümich, 2019c). Although time consuming, high-resolution depth profiles of volumetric, quantitative moisture content are more significant than the volume-averaged numbers delivered by most methods other than the NMR-MOUSE including evanescent field dielectrometry (Olmi et al., 2006). The latter method derives moisture content and the presence of salt from the dielectric properties of a wall exposed to an electric field with a frequency of about 1 GHz. The electric wave enters the wall up to about 20 mm, so the delivered moisture content is a weighted volume average across that depth range. While the measurement is fast, the depth resolution is inadequate for further analysis, because the moisture content varies significantly over the 20 mm as demonstrated with measurements of wall moisture in the Chapel of St. Mary of Chaalis Abbey (Abbeye de Chaalis) (Fig. 4). Volumetric moisture content is easy to quantify at short echo time, by simply taking the signal amplitude from a particular spot inside the wall and normalizing it to the amplitude of the signal from pure water measured with the same instrumental parameter settings.

**Figure 5:**
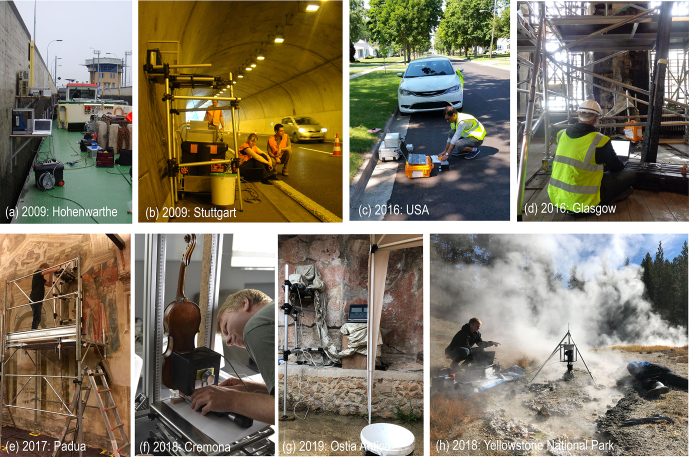
The MOUSE outside the house. **(a)** Profiling the moisture content of the gray concrete wall of the Hohenwarthe lock. **(b)** Profiling moisture in the concrete wall of the Gäubahn tunnel near Stuttgart. **(c)** Analyzing the crumb-rubber content in asphalt pavement (photo: Yadoallah Teymouri). **(d)** Assessing the fire damage of sandstone in the Mackintosh library of Glasgow. **(e)** Searching for a hidden Giotto fresco in Padua. **(f)** Measuring a depth profile though the back of a Stradivari violin in Cremona. **(g)** In search for a hidden wall painting in Ostia Antica on a rainy day. **(h)** Profiling sediment-covered biofilms at the hot springs in Yellowstone National Park.

The measurement conditions encountered in historic buildings and outdoors are often challenging to meet with equipment designed for laboratory use (Fig. 5). Examples are the presence of water, rain, or steam (Fig. 5a, g, h); passing cars (Fig. 5b, c); testing spots a few meters high (Fig. 5d, e); and treasures of outstanding value inside a guarded museum laboratory (Fig. 5f). The climate conditions can range from hot, e.g., up to 38 ${}^{\circ }$ C air temperature (Fig. 5c) or close to boiling water temperature (Fig. 5h), to cold, e.g., down to 5 ${}^{\circ }$ C (Fig. 5a). The environment may be dusty with magnetic sand particles or wet from streaming rain (Fig. 5g). In many cases, a power grid can be accessed, but in some cases, the equipment needs to be operated from a battery (Fig. 5h) or an electric power generator. Apart from the power supply, different units need to be connected with several cables at the site. These units are an NMR-MOUSE with 10 or 25 mm depth range, a precision translational stage, a fragile spectrometer console, a power supply, and a laptop computer. The electrical connectors can break during transport and assembly. The connecting cables often form ground loops that produce 50/60 Hz hum and pick up environmental electromagnetic noise. However, the latter can successfully be shielded in most cases with the help of silver-coated and electrically grounded parachute silk (Fig. 5g) or rabbit fence. Moreover, a stable scaffold finely adjustable in height and suitable to be set up on uneven ground is needed to accurately position the NMR-MOUSE at the spot of interest (Fig. 4a). All these parts along with basic tools for emergency repair are usually packed into plastic transportation boxes and shipped to the site of interest prior to the measurement campaign.

A practical point of concern in measuring high-resolution depth profiles is the proper placement of the sensitive slice parallel to the stratigraphy of the object. Assuming that the sensitive slice is 10 mm wide and 0.1 mm thick, the misalignment angle between the plane of the slice and the layers to be resolved needs to be smaller than 1 ${}^{\circ }$ (Blümich et al., 2020). With a laboratory setup, the sensitive slice and the object surface can be accurately aligned when the NMR-MOUSE is properly placed on the sample table of the lift to which the sensitive slice had been aligned by the manufacturer (Fig. 1a). But measurements in the field usually employ a translation stage without a sample table (Figs. 4a, 5f, h), so the sensor is aligned with the object surface by eye. Moreover, the minimum distance between the sensor and the object needs to be as small as possible, i.e., 1 mm or less, at the start of profiling in order to maximize the depth range into the object. Setting alignment and minimum distance is a critical part of the experiment setup. Electronic guidance for both would greatly simplify the setup procedure and improve the reproducibility of measurement.

## 4 Improving the fitness of the NMR-MOUSE for adventures outside the house

It is the accumulated experience with cultural heritage studies that suggests a number of improvements to have the NMR-MOUSE in shape for the adventures encountered when leaving the house and operating outdoors. These are the following:

1. Combine all electronics into one instrument comprising the translation stage, the NMR-MOUSE, and the transmit–receive electronics;
2. Incorporate distance and alignment sensors into the instrument;
3. Employ a stable sensor scaffold and mounting device that can be assembled quickly at the site and enable measurements at different heights.

**Figure 6:**
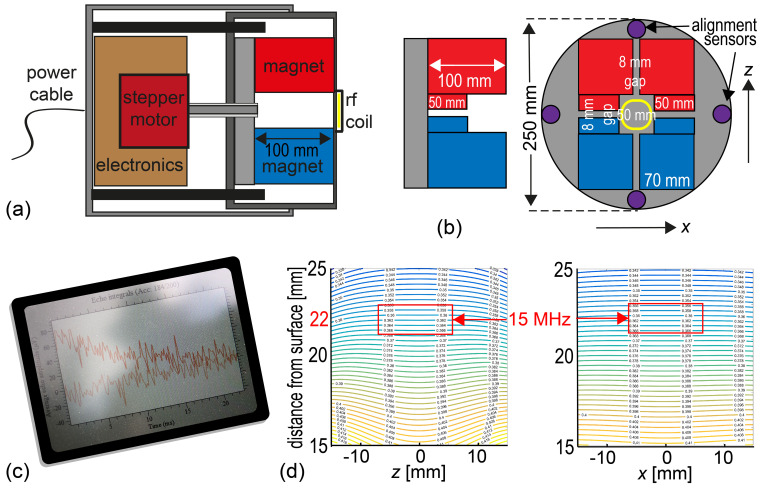
Concept of an all-in-one NMR-MOUSE for depth profiling. **(a)** Axial cross section through two telescoped tubes showing the stepper motor along with the transmit–receive electronics on the left in the outer tube and the NMR-MOUSE on the right in the inner tube. **(b)** Arrangement of magnets and alignment sensors. **(c)** The instrument should be controlled via WLAN or Bluetooth. **(d)** Calculated field map predicting the position of the sensitive slice at 22 mm above the magnet surface.

### 4.1 The all-in-one instrument

The envisioned all-in-one NMR-MOUSE for depth profiling outdoors would have a minimum number of components connected by cables (Fig. 6). The components need to be small for ease of transportation and rugged for operation outdoors. The power supply would be either a 12 V car battery or a power supply that connects to the grid or an electric generator. It hooks up to the NMR instruments (Fig. 6a) with the only cable of the setup. The instrument comprises the translation stage, the transmit–receive electronics, and the magnet (Fig. 6b). It would be operated in wireless mode via WLAN or Bluetooth from a tablet personal computer (Fig. 6c) so that it can be set up on tall scaffolds (Fig. 5e), and long measurements could be controlled and monitored from a distance including the hotel room at night.

Assuming a 250 mm inner diameter tube, a computer simulation suggests that a Profile NMR-MOUSE magnet configuration would produce a sensitive slice 22 mm above the magnet surface (Fig. 6d) with a field strength corresponding to a 15 MHz ${}^{1}$ H resonance frequency. Subtracting the space for the coil and the case, the resultant depth of access would be 15 mm, which is a reasonable working depth for many applications ranging from easel paintings to violins and frescoes. The magnet would be mounted in the inner of two telescoped tubes, which can slide in and out of the outer one under control of a precision stepper motor. The outer tube would house the stepper motor and the transmit–receive electronics and be attached to the mounting gear for depth profiling. The total length of the pipe assembly would be shorter than 300 mm.

Commercial tabletop NMR instruments employ compact transmit–receive electronics (Blümich et al., 2014; Blümich, 2016; Blümich and Singh, 2018), which, although reliable, are nevertheless too large and power-hungry for mobile use. Their state of the art has been surpassed by the development of smaller, single-chip-based magnetic resonance transceivers (Zalesskiy et al., 2014; Ha et al., 2014, 2015; Grisi et al., 2015; Chu et al., 2017; Anders et al., 2017). In particular, a small monolithic spectrometer has been developed (Bürkle et al., 2020), which uses a high-voltage CMOS processer with supply voltages up to 25 V for enhanced driving strength to combine the monolithic NMR-on-a-chip approach with macroscopic, centimeter-sized coils. This approach promises a 90 ${}^{\circ }$ pulse width of 5 µs for an echo time of 20 µs at a depth of access of 10 mm, rendering high-voltage NMR-on-a-chip transceivers well suited for use in a compact all-in-one NMR-MOUSE sensor.

### 4.2 Distance and alignment sensors

The implementation of distance and alignment sensors is a highly needed improvement over the current state of the art, where the NMR sensor has to be aligned visually parallel to the object as close as 0.5 mm (Blümich et al., 2020). The misalignment angle of the sensitive slice with the parallel layer structures of the object needs to be less than 1 ${}^{\circ }$ if the spatial resolution is to be better than 200 µm . But visual alignment parallel to an extended surface is hardly possible as one cannot see by eye the narrow gap between the magnet and the surface. Nevertheless, in practice, object and sensor have usually been aligned visually (Fig. 5f, h) with surprisingly good results in most cases but only fair reproducibility even for expert users. To enable the required reproducibility, alignment sensors need to be incorporated with the help of which the sensitive plane can be accurately aligned with the object at a fixed distance. Once aligned at a known distance, the sensor can be advanced or retracted to its starting position with the stepper motor.

**Figure 7:**
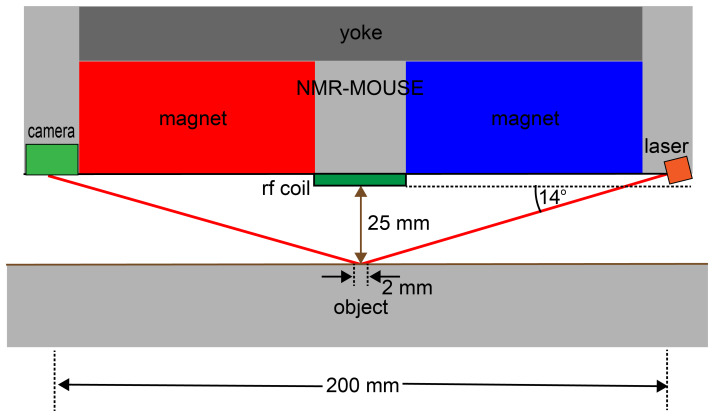
Concept of distance sensing with a laser beam at shallow incidence. If the alignment angle of the reflecting plane changes by 0.2 ${}^{\circ }$ , the reflected laser beam is displaced from the center of the detector camera by 4 mm.

Elements for alignment-sensor components can be mounted in the four spaces of the inner tube delineated by the tube's inner surface and the secants defined by the outer magnet surfaces (Fig. 5b). Different sensing principles can be considered. The surface spot to align the sensor with the object needs to be at least 1 mm wide to average effects of surface roughness. Therefore, ultrasonic distance sensors appear to be more suitable than regular laser-point distance sensors with spot widths of 10 mm vs. 70 µm , respectively. Yet commercial ultrasonic sensors measure distances within a 25 mm range with 0.75 mm reproducibility, which is an order of magnitude short of the required alignment accuracy if the sensors are 200 mm apart. Therefore, a better option are distance sensors built from lasers with a shallow angle of incidence of 14 ${}^{\circ }$ , which illuminate a 2 mm diameter spot in the center underneath the coil at 25 mm distance from the coil surface and receive the reflected light with a camera. A back-of-the-envelope calculation shows that an angle maladjustment of 0.25 ${}^{\circ }$ will shift the center of the reflected laser beam by 4 mm when laser and detector are 200 mm apart (Figs. 6, 7). This design will provide the needed accuracy and precision for alignment at a fixed distance of 25 mm. Following parallel alignment of sensitive slice and object surface, the gap between sensor and object can be shortened under control of the stepper motor before starting the acquisition of a depth profile by retracting the sensor from the wall in defined steps between measurements.

### 4.3 Operating software

To operate the equipment at locations with restricted spatial access (Fig. 5e), a wireless control strategy should be followed. The two most important operations to be under remote control are the control of the stepper motor and the data acquisition. A depth profile is typically acquired in two runs. A first profile is acquired at low spatial resolution and signal-to-noise ratio to determine the exact location and depth range of interest. Subsequently, a high-resolution profile is acquired with more scans per spot and smaller step size. Depending on the required information and the time available, either full multi-echo decays are measured for further analysis in terms of distributions of relaxation times or only the first points of the decays are recorded to determine proton density corresponding to, for example, volumetric moisture content. The measurement progress during depth profiling is usually monitored in regular intervals to catch sporadic noise interference, uninformative data, and erroneous parameter settings as budgeting time is important due to up to 2 h long acquisition times for a depth profile and limited access time in museums, historic buildings, and at excavation sites.

While operating, the NMR-MOUSE appears to be a simple endeavor to most people with a basic understanding of NMR relaxometry or MRI (Blümich et al., 2014; Johns et al., 2016); it is a true barrier to most others interested in using it for materials testing. Therefore, the operating platform should avoid NMR jargon as much as possible and relate the NMR acquisition parameters to object-specific information such as hard, soft, wet, moist, dry, moisture content, component concentration, duration of measurement, etc., which have to be entered by the operator. Prior to measurement, the proper functioning of the equipment needs to be checked, in particular the noise level and the phase angle of the transverse magnetization. The proper functioning of the equipment and potential faults should be flagged, and the receiver phase angle should be adjusted automatically if needed. Moreover, it should be possible to measure again particular depth ranges identified in previous scans. All raw data need to be saved for later access and processing in an expert mode. In addition to the data acquisition, the operating platform needs to include data processing routines that allow users to derive depth profiles with different contrast types from the acquired data, e.g., hydrogen concentration (spin density), relaxation ( $T_{2}$ ) weighted spin density ( w parameter; Blümich et al., 2014), and peak integrals and peak ratios from distributions of relaxation times as well as depth-resolved distributions of relaxation times. The latter requires access to an inverse Laplace transform algorithm. Finally, it should be possible to display the data from several measurements at the same scales for comparison and interpretation of results.

### 4.4 Mounting device and scaffold

Given the dimensions of the magnets (Fig. 6a, b), the weight of the sensor is estimated to approach 40 kg. For depth profiling, the device first needs to be positioned with high accuracy at 0.5 to 1 mm away from the surface of the object, so that depth profiles can be scanned by retracting the sensor between scans in small, preset steps of typically 0.05 to 0.25 mm. This way of scanning assures that the object is not damaged by setting a wrong depth range. The mounting device has to enable manual fine adjustment of the sensor orientation following the readings of the alignment sensors and allow for stable positioning of the sensor at various angles with high precision for long times from tens of minutes to a few hours.

**Figure 8:**
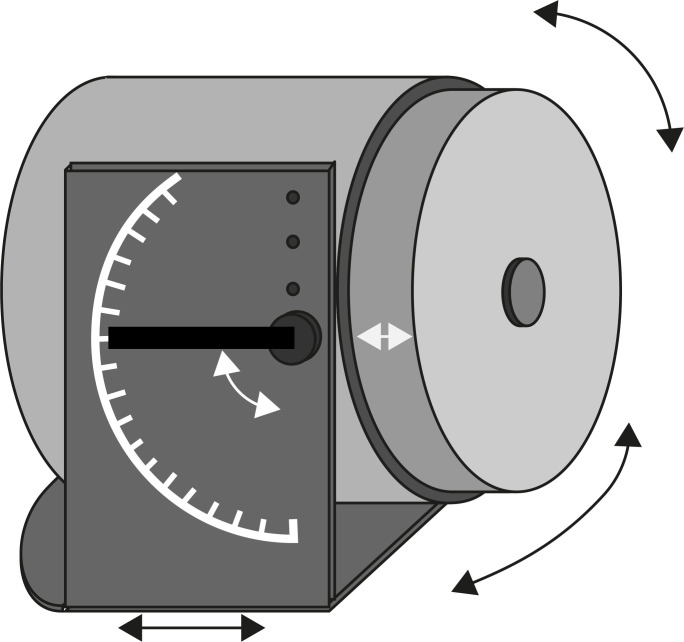
Conceptual drawing of a sensor mounting device.

A simple mounting device fulfilling these criteria would consist of a U-shaped aluminum frame (Fig. 8). It would have a flat, felt- or plastic-covered bottom without legs. Position and orientation would be adjusted manually (arrows). To access all polar angles, the polar rotation axis would be adjustable to different values in the device, and one would be able to turn the entire device upside down. To balance the sensor during depth profiling, the horizontal position of the polar rotation axis would be at the average center of gravity for a 20 mm shift range. The bottom plate would be extended at the back to provide a location for clamping the device to a scaffold or table.

For most studies outside the lab, a modular scaffold had been employed (Fig. 4a), which can be assembled from aluminum tubing with plastic joints in different ways to position the sliding table carrying the NMR-MOUSE at different heights up to about 2 m. Each of the three scaffold legs consists of two telescoped tubes, so the legs are extendable via long, threaded bolts which move the inner tubes in and out by rotating the bolt heads from the top with a cordless electric drill. While this scaffold serves its purpose and is easy to transport and set up, the scaffold is sensitive to vibrations and torsion. Moreover, it would be helpful to be able to adjust its height from the bottom and not the top and with the sensor in place, in particular when high positions need to be accessed.

## 5 Summary

Single-sided NMR relaxometry is a technique for nondestructive materials testing. Instruments like the NMR-MOUSE have been developed for use in the laboratory. Applications are found in quality control and aging of polymers and related materials, including PE pipes, PVC flooring, car tires, asphalt pavements, human skin, and food products. The dead time of the current sensors limits the detection of rapidly decaying transverse magnetization, e.g., from bound water in building materials like cement, from glass-fiber- and carbon-fiber-reinforced polymer composites in windmill wings, and varnish on paintings and musical instruments. In addition to that, in many cases, measurements need to be conducted at different depths, or complete depth profiles need to be acquired. This is the case, for example, for moisture in walls, where NMR is one if not the only method that directly measures quantitative water content without averaging over depth ranges where the moisture content significantly varies. Emerging applications in cultural heritage studies demand equipment that can easily be transported, set up, and operated. This equipment needs to be small enough and robust. The childhood and adolescence of the NMR-MOUSE equipment have been reviewed and parental advice given for preparation of outdoor measurements and survival outside the protected childhood home.

## Data Availability

Original data are no longer available due to the retirement of the leading author. Please address questions directly to the authors.

## Code availability

The magnetic field maps in Fig. 6d were calculated with COMSOL from Multiphysics GmbH, Göttingen.
